# Burnout, resilience, and empowerment among COVID-19 survivor nurses in Indonesia

**DOI:** 10.1371/journal.pone.0291073

**Published:** 2023-10-10

**Authors:** Huan-Fang Lee, Hsiang-Chin Hsu, Ferry Efendi, Vimala Ramoo, Ika Adelia Susanti

**Affiliations:** 1 Department of Nursing, College of Medicine, National Cheng Kung University, Tainan, Taiwan; 2 Department of Emergency Medicine, National Cheng Kung University Hospital, College of Medicine, National Cheng Kung University, Tainan, Taiwan; 3 Department of Emergency Medicine, College of Medicine, National Cheng Kung University, Tainan, Taiwan; 4 Faculty of Nursing, Universitas Airlangga, Surabaya, Indonesia; 5 School of Nursing & Midwifery, La Trobe University, Melbourne, Australia; 6 Department of Nursing Science, Faculty of Medicine, Universiti Malaya, Kuala Lumpur, Malaysia; 7 Faculty of Health Sciences, Universitas dr. Soebandi, Jember, Indonesia; Nazarbayev University, KAZAKHSTAN

## Abstract

The primary frontline healthcare providers who have frequent contact with COVID-19 patients are nurses. Many nurses have been infected with COVID-19 and have experienced severe emotional exhaustion and burnout. It is essential to assess nurses’ psychological health during the COVID-19 pandemic. This study aimed to analyze the factors associated with burnout, resilience, and empowerment among Indonesian COVID-19 nurse survivors. In this cross-sectional study, 182 COVID-19 survivor nurses participated from September to November 2022 with convenience sampling. An online survey using the Copenhagen Burnout Inventory (CBI), the Connor-Davidson Resilience Scale (CD-RISC), and the Psychological Empowerment Scale (PES) were used to collect data. The data were analyzed using descriptive and binary logistic regression analyses. The majority of the nurses were aged between 30–45 years (61.6%), and females (67.4%) experienced burnout. Higher resilience was found among nurses contracting COVID-19 (83.1%). In the multivariate logistic regression analysis, the absence of psychological impact (OR = 0.44, 95% CI = 0.21–0.93) is significantly related to higher burnout experience. In addition, workplace, especially in hospital (OR = 4.32, 95% CI = 1.09–17.09) was associated with resilience, and a gap time after receiving negative COVID-19 result (OR = 3.90, 95% CI = 1.27–12.03) was correlated with psychological empowerment, in our results 4–6 month after had a negative result was at higher risk. To maintain a positive psychological aspect for COVID-19 nurse survivors, it needs to implement psychological support in the workplace and ensure an appropriate workload of nurse professionals.

## Introduction

Entering the fourth year of the pandemic, the world continues to struggle with the Novel Coronavirus Disease 2019 (COVID-19) [1]. COVID-19 has been a burden and has overwhelmed the health system, especially its health workers [2, 3]. The number of confirmed cases as of the 6^th^ of January 2023 worldwide reached 657,977,736 cases with 6,681,433 deaths [4]. At the regional level, Southeast Asia became the third highest region with weekly increased cases reaching up to 13% [5]. In Indonesia, confirmed COVID-19 cases amounted to 6,723,546 with 160,683 deaths on January 9, 2023 [6]. The number of health care workers (HCWs) that have died due to the pandemic in January 2023 amounted to 2,087 [7]. COVID-19 results in high occupational exposure among health care workers, including nurses, to the disease’s spread, as well as the high need for adjustments to roles and responsibilities and professionals in clinical settings [8, 9]. According to the International Council of Nurses (ICN), the COVID-19 pandemic caused 4% of the global nursing workforce to leave. This situation raises the estimated global nursing shortage to as many as seven million [10]. The extraordinary role of nurses has a massive impact on their productivity and emotional health [11, 12].

Nurses are vulnerable to psychological distress when dealing with COVID-19 [13]. The nurses, who were primarily frontline nurses in the emergency department, experienced moderate to severe emotional exhaustion and burnout [14]. A previous study discovered that 54.2% of frontline nurses had high emotional exhaustion, 51.7% had moderate depersonalization, and 45.8% had low personal accomplishment. Another report from the United States (US) stated that 93% of HCWs were stressed and 76% were burned out [15]. Furthermore, nurses were more likely to experience burnout due to their long working hours and close contact with COVID-19 patients [16]. Several studies have found that nurses suffer from moderate to severe burnout [16–19]. Burnout not only affects their emotions but also has serious consequences for patients and the healthcare system [20], such as lower productivity, errors in clinical settings, and a lack of concern in dealing with patients [21–23]. Nurses must be able to adapt to problems and stress in order to respond to a difficult condition in COVID-19. Resilience is a psychological characteristic [24], a person develops when facing stress [25]. Resilience in nursing is an adaptive process nurses use to deal with adverse conditions and stressors in the workplace in order to create a balanced situation [26–28]. A previous scoping review discovered that nurses experienced negative resilience during the COVID-19 pandemic, exhibiting signs and symptoms such as anxiety, depression, and stress [29]. Previous research in Indonesia found that resilience is an important factor in nurses exposed to COVID-19 in hospitals [30]. It would be beneficial to help them survive and thrive during and after the COVID-19 pandemic if they have positive resilience [31].

In the same situation, the COVID-19 pandemic has caused significant clinical and social changes in healthcare systems. According to a study conducted in Jordan, survival nurses in healthcare systems have high to moderate levels of psychological empowerment [32]. The process of analyzing potential power factors in order to improve individual self-efficacy is defined as empowerment [33]. A study in the United States showed that the psychological empowerment of nurses influences better job satisfaction and organizational commitment [34]. Overall, empowerment is the cornerstone of resilience in order to avoid high levels of stressors [35].

Nurses in Indonesia have faced significant challenges during the COVID-19 pandemic. The surge in cases has put a strain on healthcare resources, leading to long working hours, high patient loads, and limited resources [36]. As a result, nurses have been at a high risk of infection and have had to deal with emotional exhaustion and burnout [37]. Despite these challenges, there is limited research on the psychological well-being of COVID-19 survivor nurses in Indonesia. While previous research has looked into the importance of resilience and psychological empowerment for nurses during the COVID-19 pandemic, it is still unclear how these factors interact and how they affect COVID-19 survivor nurses in Indonesia. Furthermore, specific factors that contribute to nurses’ empowerment and well-being, such as individual coping strategies, must be identified in order to inform interventions to support nurses in Indonesia. The purpose of this study was to analyze the factors associated with burnout, resilience, and empowerment among Indonesian COVID-19 survivor nurses.

## Materials and methods

This was a cross-sectional study that drew on primary data from an online survey. A convenience sampling method was used to collect data on demographics, burnout, resilience, and empowerment from COVID-19 survivor nurses in Indonesia. The sample size was calculated using the G-Power software version 3.1 with an a priori analysis method carried out as part of the research planning process. An α error probability of 0.05, a power of 0.8, and an effect size measured in terms of odds ratio values were chosen. The results obtained by calculating the minimum sample size were 180 respondents. There were several inclusion criteria including nurses who had recovered from COVID-19 and were working in community and clinical settings. Therefore, nurses were recruited from primary and secondary healthcare settings, including public health centers, clinics, and hospitals (both on COVID-19 wards and non-COVID-19 wards). The data collection was conducted in September-November 2022.

### Study instruments

The online questionnaire survey was divided into four sections:

1. Part I: demographic questionnaire
  The questionnaire’s profile section includes background data questions such as age, gender, marital status, and educational level. This section also includes questions about participant work arrangements and the effects of COVID-19, such as the physical and psychological impacts after COVID-19. The presence or absence of symptoms such as fever, cough, shortness of breath, or fatigue can be defined as physical impact, as can laboratory tests or imaging studies to detect evidence of infection or organ damage. Whether or not COVID-19 has a psychological impact on nurses. The psychological impact referred to here includes stress, anxiety, depression, or trauma experienced by COVID-19 survivor nurses.
2. Part II: Copenhagen Burnout Inventory scale (CBI; Kristensen, Borritz, Villadsen, and Christensen (2005))
  CBI is a 19-item scale that measures burnout in three domains: personal burnout (PB) (6 items: statement 1–6), work-related burnout (WRB) (7 items: statements 7–13), and client-related burnout (CRB) (6 items: statements 14–19). Participants rate their frequency of experiencing each symptom on a 5-point scale ranging from 0 (never) to 4 (always) for each item [38]. The result of Cronbach’s α was 0.892.
3. Part III: Connor-Davidson Resilience Scale (CD-RISC; Connor and Davidson (2003))
  The CD-RISC test assesses a person’s ability to cope with stress and adapt to adversity. It consists of 25 statements that assess five different aspects of resilience, including personal competence, instinctive trust, positive acceptance of change, control, and spiritual influences. Participants rate their agreement with each statement on a 5-point scale, from 0 (not true at all) to 4 (true nearly all of the time) [39]. The result of Cronbach’s α was 0.964.
4. Part IV: Psychological Empowerment Scale (PES; Spreitzer (1995))
  PES is a tool that assesses an employee’s sense of empowerment at work. It consists of 12 questions designed to assess four aspects of empowerment: meaning, competence, self-determination, and impact. For each item, participants rate their agreement on a 7-point scale ranging from 1 (strongly disagree) to 7 (strongly agree) [40]. The result of Cronbach’s α was 0.958.

The original English versions of the instruments were translated into the Indonesian language by two independent bilingual professionals using the back-back translation methods. To address disparities, an expert committee compared the accuracy of the translated version with the original version. To test the validity of all questionnaires, 10 nurses who met the inclusion criteria participated in the pilot study with the face validity checked by a panel of experts along with the accuracy of the translation. Based on the findings, minor changes were made to improve the questionnaire’s clarity and readability. The final questionnaire was used to collect data.

### Data collection

The research recruitment process began with socialization about research participation to Indonesian health workers via the WhatsApp (WA) group for Indonesian health workers. Nurses who met the inclusion criteria were given a brief overview of the study and asked if they would be willing to participate. An online survey with Google Form was used to collect data. Before proceeding with the survey, participants are asked to provide consent by ticking the box on the Google form. Informed consent includes information such as an explanation, purpose, participant, anonymity, and willingness to participate in the research. The participants were then asked to provide a statement that they had been infected with COVID-19, as evidenced by a coded PCR test result or a unique identification on the test result, which was subsequently utilized to match participants’ responses during data analysis. Following their explanation, participants responded to research items on burnout, resilience, and empowerment using the same Google Form. Participants were given detailed instructions on how to complete the questionnaire, and they were also informed that their participation was entirely voluntary and that they could opt out at any time.

The study followed stringent ethical rules to preserve participants’ rights, confidentiality, and privacy. To ensure confidentiality and anonymity, respondents did not provide identifying information, but were assigned a unique identifier code to link their responses. The data was kept private, accessible only to the research team, and stored securely on password-protected computers. The online data collection platform was also encrypted with SSL (Secure Sockets Layer) to prevent unauthorized access. This study was approved by the Health Research Ethics Commission of the Faculty of Nursing, Universitas Airlangga (2642-KEPK).

### Data analysis

A descriptive analysis was used to describe the demographic data of respondents. The association between demographic factors and burnout, resilience, and empowerment of COVID-19 survivor nurses was assessed using a binary logistic regression test with an odds ratio (OR) and 95% Confidence Interval (CI). The binary logistic regression assumptions were addressed, which included having a binary dependent variable, independent observations, low multicollinearity among independent factors, linearity of independent variables and log odds, and meeting the sample size criterion. SPSS version 25 was used to conduct the analysis.

## Results

### Socio-demographic characteristics of respondents

In this study, 182 nurses infected with COVID-19 were among the participants with 60% response rate from all WA group members. The results showed that the study participant was mostly aged 30–45 years (57.7%), female (65.4%), and had married status (73.6%). That was also found that most of the nurses with a bachelor’s degree (53.8%), permanent employment status (63.2%), working in a hospital (57.7%), and in COVID-19 rooms (59.0%). Most nurses have a frequency of having contracted COVID-19 once (79.7%), negative COVID-19 test results >12 months (42.3%), and an immunization history before contracting COVID-19 (62.1%). Nurses with COVID-19-infected families had up to 69.8% higher scores, while nurses who have no family/relatives died because of COVID-19 higher by 76.4%. Nurses were not affected by COVID-19 both physically and psychologically was higher (54.4% and 63.2% respectively) (Table 1).

**Table 1:** Socio-demographic characteristics of the respondents (N = 182).

Characteristics	n	(%)
**Age (years)**		
<30	71	39.0
30–45	105	57.7
>45	6	3.3
**Gender**		
Female	119	65.4
Male	63	34.6
**Marital Status**		
Single	43	23.6
Married	134	73.6
Widow/widower	5	2.7
**Education**		
Diploma Degree	74	40.7
Bachelor’s degree	98	53.8
Master’s degree	10	5.5
**Employment Status**		
Non-Permanent	67	36.8
Permanent	115	63.2
**Workplace**		
Hospital	105	57.7
Public Health Center	57	31.3
Clinic	20	11.0
**Hospital Room**		
General ward	38	36.2
Outpatient	5	4.8
COVID-19 ward	62	59.0
**Frequency of infected COVID-19**		
1	145	79.7
2	33	18.1
>2	4	2.2
**Negative COVID-19 results**		
≤3 months	25	13.7
4–6 months	27	14.8
7–9 months	26	14.3
9–12 months	27	14.8
>12 months	77	42.3
**Getting vaccinated before contracting COVID-19**		
Not	69	37.9
Yes	113	62.1
**Frequency of vaccines before contracting COVID-19**		
Never	67	36.8
Dose One	26	14.3
Dose two	42	23.1
Dosa three	43	23.6
Dose four	4	2.2
**Family/relatives infected by COVID-19**		
No	55	30.2
Yes	127	69.8
**Family/relatives who have died of COVID-19**		
No	139	76.4
Yes	43	23.6
**The physical impact of COVID-19**		
No	99	54.4
Yes	83	5.6
**The psychological impact of COVID-19**		
No	115	63.2
Yes	67	36.8
**Total**	182	100.0

### Levels of burnout, resilience, and empowerment among COVID-19 survivor nurses

After examining the levels of burnout, resilience, and empowerment of nurses who had been infected with COVID-19, it was found that most had low levels of burnout and resilience (52.75% and 57.70% respectively), while in terms of empowerment, they had more negative empowerment (55.50%) (Table 2).

**Table 2:** Descriptive analyses of nurses’ burnout, resilience, and empowerment (N = 182).

Variable	Low		High		Total
	**N**	**%**	**n**	**%**	
**Burnout**	96	52.75	86	47.25	182
**Resilience**	105	57.70	77	42.30	182
	**Negative**		**Positive**		**Total**
	**N**	**%**	**n**	**%**	
**Empowerment**	101	55,50	81	44,50	182

### Factors related to burnout, resilience, and empowerment

Binary logistic regressions were used to investigate the determinant factors of burnout, resilience, and empowerment (Table 3). The psychological impact of COVID-19 was the only significant factor related to burnout. Nurses who experienced no psychological impact from COVID-19 were less likely to experience burnout than those who experienced 0.4 times (OR = 0.44, 95% CI = 0.21–0.93) the psychological impact. Nurses without psychological impact from COVID-19 only had a 40% chance of experiencing burnout, compared to those with psychological impact. A positive relationship was also discovered between the workplace and resilience. Nurses working in regional public hospitals had 4.3 times greater resilience (OR = 4.32, 95% CI = 1.09–17.09), and those working in public health centers had an 8.7 times greater resilience (OR = 8.70, 95% CI = 2.13–35.47) of those who worked in clinics. COVID-19 survivor nurses with a negative COVID-19 result of 4–6 months were 3.9 times more likely to have positive empowerment compared to >12 months (OR = 3.90, 95% CI = 1.27–12.03).

**Table 3:** Binary logistic regression for the factors associated with burnout, resilience, and empowerment.

Variables	Burnout				Resilience				Empowerment			
	OR	Sig	95% CI		OR	Sig	95% CI		OR	Sig	95% CI	
			Lower	Upper			Lower	Upper			Lower	Upper
**Age (years)**												
<30	1.16	0.89	0.15	9.19	5.20	0.19	0.43	62.41	2.09	0.51	0.23	19.22
30–45	1.62	0.63	0.23	11.44	3.45	0.31	0.32	37.61	1.14	0.90	0.14	9.29
>45	1.00				1.00				1.00			
**Gender**												
Female	0.93	0.84	0.43	1.99	1.91	0.10	0.88	4.17	1.08	0.83	0.52	2.27
Male	1.00				1.00				1.00			
**Marital Status**												
Single	1.52	0.72	0.15	15.51	1.91	0.60	0.17	21.63	0.21	0.16	0.02	1.82
Married	0.57	0.61	0.06	5.03	3.49	0.30	0.33	36.67	0.43	0.41	0.06	3.25
Widow/widower	1.00				1.00				1.00			
**Education**												
Diploma Degree	0.82	0.81	0.17	4.08	0.85	0.84	0.17	4.27	0.22	0.06	0.05	1.09
Bachelor’s degree	0.57	0.48	0.12	2.70	0.55	0.47	0.11	2.71	0.26	0.09	0.06	1.21
Master’s degree	1.00				1.00				1.00			
**Employment Status**												
Non-Permanent	0.56	0.16	0.25	1.26	1.17	0.70	0.53	2.60	1.09	0.82	0.50	2.39
Permanent	1.00				1.00				1.00			
**Workplace**												
Hospital	1.05	0.93	0.31	3.56	4.32	0.03*	1.09	17.09	1.24	0.72	0.37	4.12
Public Health Center	0.56	0.37	0.16	1.97	8.70	0.001*	2.13	35.47	2.20	0.20	0.65	7.44
Clinic	1.00				1.00				1.00			
**Frequency of infected COVID-19**												
1	0.37	0.43	0.03	4.45	3.11	0.39	0.23	41.24	0.65	0.74	0.05	8.76
2	0.54	0.64	0.04	6.97	2.43	0.51	0.17	34.54	0.53	0.64	0.04	7.69
>2	1.00				1.00				1.00			
**Negative COVID-19 results**												
≤3 months	0.59	0.34	0.20	1.76	1.05	0.93	0.35	3.13	1.59	0.40	0.54	4.63
4–6 months	1.26	0.69	0.39	4.06	0.83	0.75	0.27	2.59	3.90	0.02*	1.27	12.03
7–9 months	0.61	0.38	0.20	1.84	0.49	0.23	0.15	1.56	1.31	0.61	0.46	3.76
9–12 months	0.37	0.06	0.13	1.05	1.01	0.98	0.37	2.76	2.62	0.06	0.96	7.11
>12 months	1.00				1.00				1.00			
**Getting vaccinated before contracting COVID-19**												
No	0.34	0.12	0.08	1.33	0.72	0.67	0.16	3.22	2.44	0.19	0.63	9.34
Yes	1.00				1.00				1.00			
**Frequency of vaccines before contracting COVID-19**												
Never	3.10	0.40	0.22	44.00	0.63	0.73	0.05	8.33	0.79	0.85	0.07	8.92
Dose One	1.12	0.94	0.07	17.51	0.32	0.41	0.02	4.89	3.16	0.38	0.24	41.30
Dose two	1.75	0.68	0.12	26.25	0.31	0.39	0.02	4.51	1.00	0.99	0.08	12.59
Dosa three	1.03	0.98	0.07	14.73	0.45	0.55	0.03	6.08	1.49	0.75	0.12	17.79
Dose four	1.00				1.00				1.00			
**Family/relatives infected by COVID-19**												
No	0.87	0.73	0.39	1.95	2.24	0.06	0.98	5.11	1.22	0.62	0.55	2.67
Yes	1.00				1.00				1.00			
**Family/relatives who have died of COVID-19**												
No	0.47	0.09	0.20	1.12	1.31	0.53	0.56	3.07	0.97	0.94	0.43	2.18
Yes	1.00				1.00				1.00			
**The physical impact of COVID-19**												
No	0.54	0.09	0.27	1.10	1.77	0.13	0.84	3.69	1.34	0.42	0.66	2.73
Yes	1.00				1.00				1.00			
**The psychological impact of COVID-19**												
No	0.44	0.03*	0.21	0.93	0.95	0.90	0.45	2.03	0.81	0.58	0.40	1.67
Yes	1.00				1.00				1.00			

## Discussion

This study aimed to analyze the determinant factors of burnout, resilience, and psychological empowerment among COVID-19 survivor nurses. The results showed that most nurses between the ages of 30 and 45 experience burnout. This correlates with a previous study that nurses aged 42 left their jobs due to burnout [41]. Burnout levels were higher in older nurses as they correlated with stress and level of professional identity [42]. Occupational burnout can be defined as an individual’s inability to achieve satisfactory outcomes at work, aversion to work, apathy, and an indifferent attitude [43]. Furthermore, female nurses had the highest percentage of burnout. A study conducted in China and Taiwan supports this study that burnout is particularly prevalent among female nurses [44]. They are more likely to suffer from symptoms of depression and emotional exhaustion than male nurses [45]. Female nurses have been shown to experience higher levels of stress when dealing with patients affected by COVID-19 [46]. Nurses between the ages of 30 and 45, as well as female nurses, are more likely to experience burnout. However, regression analysis revealed that these variables were not predictors/factors of burnout in this study.

There was a link correlation between the psychological impact of COVID-19 and burnout in COVID-19 survivor nurses. Nurses who did not experience any psychological impacts from COVID-19 were less likely to experience burnout. This finding is consistent with previous studies that have suggested the psychological impact of COVID-19 contributes to burnout [47, 48]. Nurses have to act quickly in dealing with COVID-19, and the workload is heavy [49]. Its consequences include an increased level of burnout syndrome. In addition, the COVID-19 pandemic has negative psychological impacts such as post-traumatic stress, primarily among nurses [50]. A previous study showed that almost all health professionals (90.4%) need psychological care during COVID-19 which should be provided by work centers [49]. During the pandemic, nurses in Indonesia face unique challenges, such as limited resources and heavy workloads, which can exacerbate fatigue and negatively impact mental health. Furthermore, a lack of public awareness, public ignorance, a lack of role models, stigma, and discrimination have all had a negative impact on nurses’ mental health and well-being [37]. As mentioned above, it is essential to support nurses with positive psychological change during the COVID-19 pandemic with high challenges and stressful situations.

COVID-19 survival nurses working in public regional hospitals were more likely to have higher resilience compared to public health centers and clinics. The previous study found that resilience is related to nurses’ ability to adapt to workplace challenges and minimize the negative effects of occupational demands [51]. A study conducted in one of Indonesia’s public hospitals found that nurses there have high resilience, reaching 90.4% [52]. Nurses with high resilience can help them respond to workplace stress and complex conditions [53]. Another study reported that fear of becoming infected, intention to leave work, and having a positive COVID-19 test were associated with lower resilience scores during the COVID-19 pandemic. According to the study, nurses who reported organizational support were significantly more resilient than those who did not [31]. We contended that nurses in public hospitals receive positive support from their employers and feel safe and meaningful. Furthermore, the public regional hospital is financially more stable. In April 2020, the Indonesian government announced a bonus for healthcare workers involved in the COVID-19 response, including nurses. The government also provided additional funds to hospitals and healthcare facilities to help them prepare for the pandemic [54, 55]. A meta-analysis study emphasized the importance of nurse support programs on job satisfaction among hospital nurses [56]. According to a study conducted in a major public hospital, 67.8% of nurses working there were satisfied with their jobs [57]. So, it has an impact on the hospital’s ability to ensure job satisfaction of nurses. However, no studies compare the resilience of COVID-19 survival nurses working in different settings.

Furthermore, this study examined the determinant of empowerment for COVID-19 survival nurses. The findings revealed that nurses with negative COVID-19 results for 4–6 months were more likely to have positive psychological empowerment than nurses with negative results for >12 months. Negative results of COVID-19 increase psychological effects such as emotional and physical dimensions of well-being, mental disorders, housing situation, partnership, and future expectations [58]. There are few studies examining the relationship between COVID-19’s negative results and empowerment. We assumed that nurses who had negative COVID-19 results for 4–6 months would have positive empowerment as a result, which was conducted on matured nurses. It has to do with their ability to adjust and adapt to changing circumstances during the COVID-19 pandemic. They may feel more relieved and confident in their health status, leading to higher levels of psychological empowerment. Natural immunity following COVID-19 infection, as we know, can provide good protection against disease symptoms and hospitalization with an average effectiveness of at least 88% up to 10 months after infection [59, 60]. Overall, the literature results indicate that perceived health status, clinical competency, online support programs, mental health conditions, and resilience influence nurses’ psychological empowerment during the COVID-19 pandemic [61, 62]. However, more research is needed to determine the specific reasons for this finding. Furthermore, more intensive support in nurse coping is required to strengthen psychological empowerment among nurses by providing psychological support to deal with the negative effects of COVID-19.

There are several limitations to this study. The primary limitation of this study is its cross-sectional design. Therefore, the causal relationship cannot be examined. Because this study used only selected participants, in addition, those who participated may be nurses with characteristics of high resilience, high empowerment and low burnout. Then there is a possibility of error when conducting convenience sampling and online surveys, convenience sampling may introduce bias in the sample selection process. Finally, because this is an online survey, the study’s bias cannot be ignored. The research’s results provide important perspectives for the specific population and situation researched. Because of the limited sample size, the results may solely reflect the features and experiences of the unique participants. When extending these findings to larger populations, however, caution is advised. More study with diverse samples and contexts is needed to increase generalizability. Aside from those limitations, the results of this study help to inform determinants of burnout, resilience, and psychological empowerment among COVID-19 survival nurses in Indonesia.

## Conclusion

Among COVID-19 survivor nurses, our results revealed significant factors related to burnout, resilience, and empowerment. The psychological impact of COVID-19, such as stress, anxiety, depression, or trauma is linked to burnout among nurses. It contributes to increased workplace burnout. There was a positive correlation between workplace and resilience among nurses. Furthermore, negative COVID-19 results were linked to psychological empowerment. This suggests that developing resilience may be an effective strategy for assisting nurses in coping with the challenges of the pandemic and reducing the negative consequences on their well-being. To improve resilience, empowerment, and burnout prevention among COVID-19 survivor nurses, psychological support in the workplace is provided, with a focus on improving job satisfaction and the quality of health outcomes. Additionally, factors related to burnout, resilience, and empowerment must be addressed, with a focus on the demographic distribution of respondents. Future research could use qualitative or mixed-methods research to gain a more comprehensive understanding of the phenomenon under investigation.
